# Benefits of treatment with favipiravir in hospitalized patients for COVID-19: a retrospective observational case–control study

**DOI:** 10.1186/s12985-021-01577-1

**Published:** 2021-05-25

**Authors:** Anıl Ucan, Pamir Cerci, Serdar Efe, Hakan Akgun, Ahmet Ozmen, Aysel Yagmuroglu, Muzaffer Bilgin, Deniz Avci

**Affiliations:** 1 Department of Internal Medicine, Eskisehir City Hospital, 71 Evler Neighborhood, Çavdarlar Street, 26080 Odunpazarı, Eskişehir Turkey; 2 Division of Immunology, Department of Internal Medicine, Eskisehir City Hospital, 71 Evler Neighborhood, Çavdarlar Street, 26080 Odunpazarı, Eskişehir Turkey; 3 Division of Intensive Care, Department of Internal Medicine, Eskisehir City Hospital, 71 Evler Neighborhood, Çavdarlar Street, 26080 Odunpazarı, Eskişehir Turkey; 4 Department of Thoracic Medicine, Eskisehir City Hospital, 71 Evler Neighborhood, Çavdarlar Street, 26080 Odunpazarı, Eskişehir Turkey; 5 Department of Infection Diseases, Eskisehir City Hospital, 71 Evler Neighborhood, Çavdarlar Street, 26080 Odunpazarı, Eskişehir Turkey; 6 Department of Microbiology, Eskisehir City Hospital, 71 Evler Neighborhood, Çavdarlar Street, 26080 Odunpazarı, Eskişehir Turkey; 7 Department of Biostatistics, Faculty of Medicine, Eskisehir Osmangazi University, Meşelik Kampüsü Büyükdere Mah. Prof. Dr. Nabi Avcı Bulvarı No: 4, 26040 Odunpazarı, Eskişehir Turkey; 8 Department of Internal Medicine, Kayseri City Hospital, Muhsin Yazıcıoğlu Bulvarı No: 77, 38080 Kocasinan Kayseri, Turkey

**Keywords:** Severe acute respiratory syndrome coronavirus 2, Pneumonia, Favipiravir, Hydroxychloroquine, Antiviral therapy

## Abstract

**Background:**

Although more than a year past since COVID-19 was defined, there is no specific treatment yet. Since COVID-19 management differs over time, it is hard to determine which therapy is more efficacious. In this study, we aimed to evaluate the efficacy of the regimen with Favipiravir (FPV) and determine if the timing of FPV addition offers any improvement.

**Methods:**

A retrospective observational case-controlled cohort study was performed between March and September 2020, including adults with COVID-19 in a single-center in Turkey. We categorized patients into age-sex matched three groups, group 1 (n = 48) and group 2 (n = 48) included patients treated with the combination of FPV plus Hydroxychloroquine (HQ) early and late, respectively. Group 3 (n = 48) consisted of patients on HQ monotherapy. In Group 2, if the respiratory or clinic condition had not improved sufficiently, FPV was added on or after day 3.

**Results:**

We found that starting FPV early had an impact on PCR negativity and the progression of the disease. 'No progression' was defined as the absence of a new finding in the control radiological examination and the absence of accompanying clinical deterioration. Also, the decrease in C-reactive protein (CRP) was greater in Group 1 than Group 3 (*p* < 0.001). However, we found that early initiation of FPV treatment did not have a positive effect on the estimated survival time.

**Conclusions:**

According to this retrospective study results, we believe that for better clinical outcomes, FPV treatment should be started promptly to enhance antiviral effects and improve clinical outcomes.

## Introduction

The SARS-CoV-2 virus, most of its clinical manifestations causing a respiratory illness with non-characteristic symptoms, was observed in a group of patients with unknown cause pneumonia in Wuhan, China, in December 2019 [1]. By the time, the cause of this disease is understood to be a new coronavirus. It was temporarily named the new coronavirus 2019 (nCoV) by the World Health Organization (WHO). In February 2020, the emerging clinical manifestation was redefined as “COVID-19” (coronavirus disease 2019). Since the beginning of the pandemic, the new coronavirus has caused over 79 million reported cases and more than 1.7 million deaths worldwide, as of December 29, 2020 [2].

In COVID-19, there is an incubation period of approximately 5.2 days between the appearance of symptoms and exposure to the drug [3]. Fatigue, fever, cough, and shortness of breath are the most frequent symptoms and clinical presentations of the disease. About 15% of patients have a severe illness requiring oxygen supply, and about 5% respiratory failure, acute respiratory distress syndrome (ARDS), there may be fatal consequences resulting in sepsis and septic shock, thromboembolism, and/or multiorgan failure [4].

Since COVID-19 management differs over time it is hard to determine which therapy is more efficacious. In a previous study, Favipiravir (FPV), which can be a good treatment alternative in many types of RNA viruses, has demonstrated its antiviral effect on RNA-dependent RNA polymerase gene inhibition in vitro and in vivo [5]. We assumed that this drug has similar efficacy in the 2019-nCoV virus, a beta-coronavirus, whose genome sequence is defined as single-stranded RNA. FPV has been presented as an effective drug for all subtypes of influenza viruses that resist neuraminidase and M2 inhibitors as a potent and selective RNA polymerase inhibitor [5]. After the positive effects of survival in the EBOLA virus outbreak in 2014, safety and efficacy studies have shown that FPV can also be useful on SARS-CoV-2. It has been found that the drug has positive effects on chest images and early viral clearance [6, 7]. In a multicenter randomized controlled clinical trial supporting this study's results, FPV treatment for COVID-19 was observed to have a positive effect (from 55 to 71%) over 7-day clinical recovery rates [6]. It was recommended for COVID-19 in the guidelines published by the Turkish Ministry of Health's Scientific Committee [8].

In this study, we aimed to evaluate the efficacy of the regimen with FPV and to find out if the timing of FPV addition offers any improvement. These findings will help enlighten clinicians for the clinical treatment of SARS-CoV-2 infection.

## Methods

### Study participants

A retrospective observational case-controlled cohort study, including adults with laboratory-confirmed COVID-19 in a single center in Turkey, was performed between March 2020 and September 2020 at Eskişehir City Hospital. All patients who were treated and followed by the researchers at our hospital were included in the study. The patient's medical records were gathered in the hospital's electronic database. Patients groups of the study were determined according to the recommendations of the guidelines published by the Turkish Ministry of Health's Scientific Committee updated on different dates. All patients were evaluated in terms of their epidemiological and demographic characteristics, laboratory and radio-diagnostic tests, treatment features, and outcomes. Medical ethics committee approval was obtained prior to the study. Routine blood tests were recorded of the patients at first hospitalization; Complete Blood Count (CBC), C-reactive protein (CRP), Ferritin, D-Dimer, liver and kidney function tests, SARS-CoV-2 nucleic acid, and other laboratory results were compared to subsequent simultaneous controls. The primary endpoint was reaching PCR negativity. The secondary endpoints were the rate of admission to an ICU and the rate of death. Clinical classification of the mild, moderate, severe, and critical patients was performed according to the WHO guideline [9].

### Radiology measures

Chest CT scans were conducted on day 1 and the 14th day of hospitalization. In the study, patients with confirmed COVID-19 pneumonia, serial chest CT scans were recorded and reviewed by three physicians. We use COVID-19 Reporting and Data System (CO-RADS) for standardized assessment of pulmonary involvement and classification of COVID-19 patients' findings in our study. The CO-RADS classification evaluates the suspected pulmonary involvement of COVID-19 on a scale from 0 (not interpretable) to 5 (proven) [10]. This classification is specifically designed for use in patients with moderate to severe symptoms of COVID-19.

### Eligibility criteria

In terms of eligibility criteria, we evaluated all patients admitted to three groups. Inclusion criteria were positive SARS-Cov-2 virus reverse transcription PCR (RT-PCR) test, age > 18 years old, no difficulty in swallowing the pills, and hospitalized in COVID-19 clinics and ICU. The exclusion criteria included the following: patients who do not have PCR testing or inadequate diagnosis, patients with mental disorders, previous history of allergic reactions, patients with elevated ALT/AST (> 6 × upper limits of normal range) or with chronic liver disease and cirrhosis, patients with a history of all lung diseases other than COPD, pregnant or lactating women; women of childbearing age with a positive pregnancy test and breastfeeding.

### Treatments

We informed our staff at our medical center for FPV, which is recommended by the COVID-19 treatment algorithms in guidelines published by the Turkish Ministry of Health's Scientific Committee for moderate to severe disease in April 2020 and asymptomatic to the severe disease for all definite cases in October 2020. Patients were divided into three groups of treated with FPV / (HQ) combination early (Group1) versus late (Group 2) and HQ only (Group 3). The treatment regime was; FPV 1600 mg twice the first day, followed by 600 mg twice daily, for the following days for five days at standard treatment and HQ 400 mg for five days. In Group 2, we treated HQ initially; if the respiratory or clinic condition had not improved sufficiently, FPV was added on or after day 3. In both groups, patients took supportive treatments such as oxygen inhalation, intravenous and oral hydration, antibiotics, antipyretics, and analgesics when needed.

### Procedures

The diagnosis, monitoring, and treatment for COVID-19 were based on the guidelines published by the Turkish Ministry of Health's Scientific Committee on COVID-19 [8]. Sampling was taken as a throat swab at seven days intervals in our study until a negative result was seen (1, 5, 7, 14, 21, and 28). All samples were tested in our biosafety level-2 (BSL-2) laboratory facility with full personal protective equipment. Viral analyses and real-time (Q) PCR experiments were applied to the sample of the patient. The RNA was isolated using vNAT solution (Bioeksen, Istanbul, Turkey). For all reactions, Rotor-Gene Q (Qiagen) and LightCycler 480 (Roche) instrument, and Biospeedy SARSCoV-2 RT-qPCR kit (Bioeksen) were used. The kit is performed in one step with targeting the RdRp (RNA-dependent RNA polymerase) gene fragment reverse transcription (RT) and rt PCR (QPCR) (RT-QPCR). The data was analyzed using Rotor-Gene Q and LightCycler 480 Software. Bio-Speedy kit used (which Bioeks, Istanbul, Turkey), Coronavirus Disease 2019 (Covidien-19) that leads to disease pandemics of SARS-CoV-2 (2019-NCover) is used for the detection of the virus. The kit is applied to nucleic acid isolates from nasopharyngeal aspirate/lavage, bronchoalveolar lavage, nasopharyngeal swab, oropharyngeal swab, and sputum samples. The shape of the growth curves obtained from the FAM / HEX channels is examined. Non-sigmoidal curves are recorded as negative. The threshold cycle number (Cq) is calculated. The sensitivity and specificity of the Biospeedy kit is 98.7–100%, respectively.

### Evaluation of response to treatment

Laboratory parameters were evaluated on the day of hospitalization, and routine examinations were performed on the 7th day. Values were compared numerically. While evaluating the effectiveness of the treatment in the patients, it was interpreted by comparing the computed tomography (CT) scans with the clinical conditions after the 14th day of hospitalization. The PCR test was checked on the 7th day of the first test to interpret the drug's effectiveness, and it must be negative.

### Ethics

The study was approved by the Turkish Ministry of Health and the Ethics Committee of the Faculty of Medicine at Eskişehir Osmangazi University (approval number 2020/259) and was carried out in accordance with the Declaration of Helsinki principles and all applicable regulations.

### Statistical analysis

Descriptive data are presented as either means or median for continuous variables, frequencies and percentages are reported for categorical variables. We reported continuous data as mean ± standard deviation, or median, and minimum–maximum according to their distribution. Pearson X2 test is used to assessing the associations in categorical variables. The pre and post values of the parameters were compared using the paired sample *t* test and two-way ANOVA. Tukey's test and Games Howell tests were preferred as posthoc tests. The time to clinical improvement was portrayed by Kaplan–Meier plot and compared with a log-rank test. Hazard ratios with 95% confidence intervals were calculated by means of the Cox proportional-hazards model. A *p*-value of < 0.005 is considered as statistical significance. The statistical analysis of the study was performed with SPSS software (Statistical Package for the Social Sciences, version 22.0, SPSS Inc, Chicago, IL).

## Results

### Baseline characteristics

A total of 144 laboratory-confirmed COVID-19 patients who underwent randomization and had started treatment between March and September 2020 were enrolled. Twelve patients were excluded due to a lack of adequate imaging and laboratory findings. Considering all groups, % 55.5 were male. In Groups 1, 2, and 3, median ages were 58.5 (range, 50–65) years, 59.5 (range 45–67) years, 56 (45–66.75) years, respectively. There was no substantial and statistically difference in basic characteristics between groups.

The disease classification of the patients was evaluated with the Clinical Management of COVID-19 Interim Guidance published by WHO. Patients were divided according to disease severity into five as asymptomatic, mild, moderate, severe, and critical [9]. In Group 3, 13 (%27.1) patients were asymptomatic and 35 (%72.9) patients presented with mild to moderate symptoms. In Group 1 and 2, patients with severe and critical severity were 12 (%25.5) and 8 (%16.7), respectively. When the comorbid conditions of the patients were compared, no statistical difference was found between the groups in terms of having Diabetes, Cardiovascular Disease, Hypertension, COPD diseases. The number of patients with COPD was similar in all three groups. Also, the smoking of patients did not show a statistical difference between the three groups (*p* = 0.666). It was noted that headache was less in Group 3 (for Group 1 33.3%, and for Group 2 39.6% compared to 8.3%). Nausea and vomiting were statistically significantly higher in Group 2 when comparing Group 3 (*p* = 0.020) (Table 1).

**Table 1:** Clinical conditions of the patients at admission (Group 1: Early treatment with FPV and HQ, Group 2: Late treatment with FPV and HQ, Group 3: Treatment with only HQ)

	Group 1	Group 2	Group 3	*p*-value
Symptoms before admission to hospital (mean [SD] days)	4.73 (2.447)	3.73(2.141)	2.667 (2.628)	NS
Drugs use				
Anti-hypertensive	20 (41.7%)	17 (35.4%)	20 (41.7%)	NS
Anti-diabetic	19 (39.6%)	13 (27.1%)	14 (29.2%)	NS
Symptoms in admission to hospital				
Fatigue	25(52.1%)	35(72.9%)	22 (45.8%)	NS
Fever	19 (39.5)	7 (%14.5)	7 (%14.5)	NS
Cough	30(62.5%)	39 (81.2%)	20 (41.7%)	NS
Shortness of breath	28 (58.3%)	24 (50.0%)	6 (12.5%)	NS
Nasal congestion	2 (4.2%)	2 (4.2%)	1 (2.1%)	NS
Sore throat	9 (18.8%)	10 (20.8%)	8 (16.7%)	NS
Nausea and vomiting	4 (8.3%)	13 (27.1%)^a^	5 (10.4%)^a^	**0.020**^a^
Headache	16^b^ (33.3%)	19^c^ (39.6%)	4^b,c^ (8.3%)	**0.001**^b^ **0.001**^c^
Anosmia	4(8.3%)	1(2.1%)	0 (0.0%)	NS
Diarrhea	5(10.4%)	6 (12.5%)	5 (10.4%)	NS
FPV received after symptoms onset (mean [SD] days)	4.73(2.447)	8.60 (3.654)		** < 0.001**

Statistically significant values are presented in bold. Superscript letters define the significant *p*-values of pairwise comparisons.

*FPV* Favipiravir; *NS*: non-significant

^a^ denotes the comparison of patients with nausea and vomiting in Group 2 vs. Group 3

^b^ denotes the comparison of patients with headache in Group 1 vs. Group 3

^c^ denotes the comparison of patients with headache in Group 2 vs. Group 3

### Laboratory tests

The laboratory parameters on the hospitalization day and the routine examinations performed on the 7th day of hospitalization were compared, as shown in Table 2. We investigated and found a result of whether there is a difference between the groups in the CRP and Ferritin values. But we also found other important laboratory parameters such as lymphocyte and D-dimer changes did not differ statistically between the three groups (Table 3).

**Table 2:** Comparison of Laboratory Results Before and After the Treatment in Groups (Group 1: Early treatment with FPV and HQ, Group 2: Late treatment with FPV and HQ, Group 3: Treatment with only HQ)

Changes in variables	Group 1		Group 2		Group 3	
	Mean ± SD	*p*	Mean ± SD	*p*	Mean ± SD	*p*
CRP	15.2 ± 39.2	0.077	20.2 ± 63.8	**0.045**	16.2 ± 68.2	0.113
Lymphocyte	− 478.9 ± 930.0	0.066	− 275.5 ± 643.6	**0.007**	55.1 ± 697.9	0.595
D-Dimer	− 0.74 ± 2.06	0.287	− 0.96 ± 4.71	0.212	0.09 ± 0.63	0.371
Ferritin	− 106.6 ± 280.5	0.287	52.5 ± 254.4	0.276	26.5 ± 487.5	0.739
Cr	− 4.91 ± 8.97	0.052	0.01 ± 0.64	0.902	− 0.68 ± 4.94	0.113
AST	10.4 ± 14.7	**0.003**	4.2 ± 89.8	0.753	8.2 ± 37.5	0.148
ALT	− 8.2 ± 21.2	0.208	− 4.3 ± 93.7	0.757	− 6.8 ± 28.0	0.101

Statistically significant values are presented in bold. The median values given are the numerical results calculated by the difference of consecutive tests. A negative value means decrease whereas a positive value indicates an increase. The pre and post values of the parameters were compared using the paired sample *t* test and two-way ANOVA

*ALT* alanine aminotransferase; *AST* aspartate aminotransferases; *CRP* c-reactive protein; *Cr* creatinin; *NS*: non-significant

**Table 3:** Comparison of laboratory results before and after the treatment between groups (Group 1: Early treatment with FPV and HQ, Group 2: Late treatment with FPV and HQ, Group 3: Treatment with only HQ)

	Compare groups	Mean difference	SE	*p*	95% CI
CRP	Group 2–Group 3	37.0	9.9	**0.001**	13.3–60.6
	Group 1–Group 2	13.9	12.5	0.512	− 15.6–43.7
	Group 1–Group 3	50.9	10.2	** < 0.001**	26.5–75.2
Lymphocyte	Group 2–Group 3	16.8	166.3	0.994	− 425.0–391.4
	Group 2–Group 1	253.0	129.8	0.131	− 56.8–562.7
	Group 3–Group 1	236.2	152.5	0.286	− 143.7–616.1
D-Dimer	Group 2–Group 3	0.41	0.72	0.837	− 1.34–2.16
	Group 2–Group 1	0.83	0.58	0.333	− 0.58–2.25
	Group 3–Group 1	0.42	0.88	0.881	− 1.68–2.53
AST	Group 2–Group 3	20.1	7.3	**0.022**	2.42–37.7
	Group 1–Group 2	2.4	8.2	0.952	− 17.1–22.0
	Group 1–Group 3	22.5	5.5	** < 0.001**	9.4–35.6
ALT	Group 2–Group 3	10.2	8.3	0.441	− 9.8–30.3
	Group 1–Group 2	3.9	8.3	0.887	− 16.0–23.8
	Group 1–Group 3	14.1	6.4	0.083	− 1.5–29.8
Ferritin	Group 2–Group 3	240.2	90.0	**0.030**	20.2–460.2
	Group 1–Group 2	119.1	151.3	0.712	− 244.7–482.8
	Group 1–Group 3	359.3	137.5	**0.032**	25.6–693.1
Cr	Group 3–Group 2	2.39	1.17	0.138	− 0.67–5.44
	Group 1–Group 2	0.25	0.39	0.804	− 0.69–1.18
	Group 3–Group 1	2.15	1.22	0.212	− 0.98–5.27

Statistically significant values are presented in bold. Tukey's test and Games Howell tests were preferred as posthoc tests

*ALT* alanine aminotransferase; *AST* aspartate aminotransferases; *CRP* c-reactive protein; *Cr* Creatinin

### Primary and secondary outcomes

**Fig. 1:**
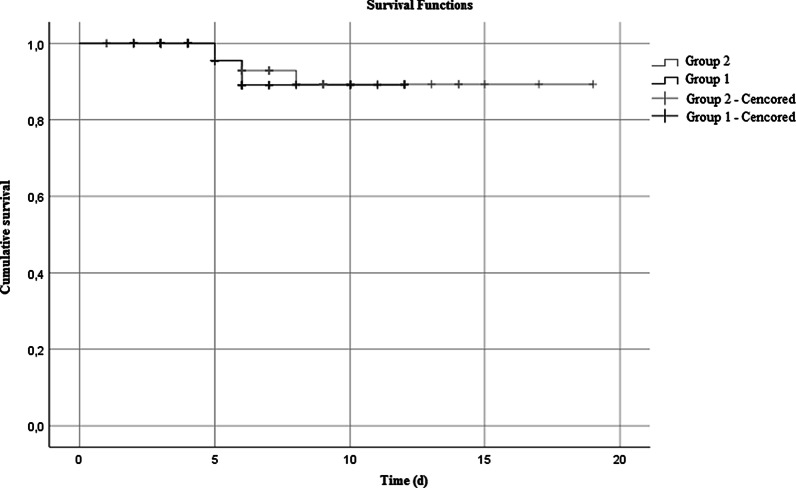
Kaplan–Meier analysis for survival with FPV treatment, Group 1: Early treatment with FPV and HQ, Group 2: Late treatment with FPV and HQ

Patients were classified according to clinical outcome from admission [9]. It was observed that the rate of development of ARDS and the rate of Intensive Care admission was significantly lower in patients who were treated with FPV (Group 1 and 2) compared to patients who were treated with HQ (Group 3) (Table 4). We conduct a Kaplan–Meier analysis of length for survival between Group 1 and 2, which were presented in Fig. 1. The mean time of estimated survival time for the patients in Group 1 was 11.3 d (IQR: 10.3–12.2 and Group 2 was 17,6 d (IQR: 16.3–18.9 (*p* = 0.810). Comparing the patient's estimated survival time in Group 1 to Group 2, we found it wasn't significantly longer than Group 2 patient's time. There was no mortality in Group 3 therefore a Kaplan–Meier survival analysis was not calculated for this group of patients.

**Table 4:** Clinical outcomes of patients(Group 1: Early treatment with FPV and HQ, Group 2: Late treatment with FPV and HQ, Group 3: Treatment with only HQ)

Clinic outcomes	Group 1	Group 2	Group 3	*p*-value
ARDS	6^a^ (12.5%)	10^b^ (20.8%)	0^a,b^ (0.0%)	**0.005**^a^ **0.005**^b^
ICU	13^c^ (27.1%)	13^d^ (27.1%)	1^c,d^ (2.1%)	**0.001**^c^ **0.001**^d^
Intubation	2 (4.2%)	4 (8.3%)	0 (0.0%)	NS
Death	2 (4.2%)	4 (8.3%)	0 (0.0%)	NS

Statistically significant values are presented in bold. Superscript letters define the significant *p*-values of pairwise comparisons. Tukey's test and Games Howell tests were preferred as posthoc tests

*ARDS* acute respiratory distress syndrome; *ICU* ıntensive care unit

^a^ denotes the comparison of patients with ARDS in Group 1 vs. Group 3

^b^ denotes the comparison of patients with ARDS in Group 2 vs. Group 3

^c^ denotes the comparison of patients with ICU in Group 1 vs. Group 3

^d^ denotes the comparison of patients with ICU in Group 2 vs. Group 3

**Fig. 2:**
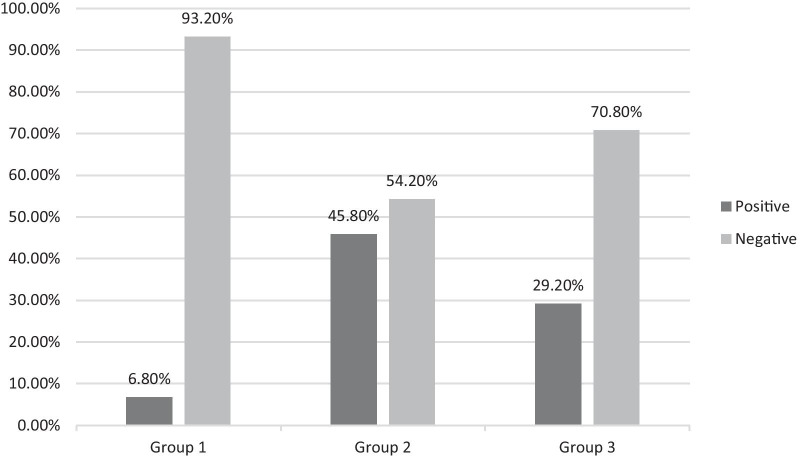
Second PCR results after treatment; Group 1: Early treatment with FPV and HQ, Group 2: Late treatment with FPV and HQ, Group 3: Treatment with only HQ

Second PCR test negativities and study groups were compared (Fig. 2). Statistically, Group 1 was superior to Group 2 (*p* < 0.001) and Group 3 in terms of PCR negativity (*p* < 0.001). In order to measure the relationship between the clinical conditions of the patients and drugs, the patients were examined in three groups. Here, pre-treatment and post-treatment evaluation of the radiological image together with clinical evaluation, was chosen as the method. 'No progression' was defined as the absence of a new finding in the control radiological examination and the absence of accompanying clinical deterioration. When the patients' no progression status is examined; 54.2% (n = 26/48) of the patients in Group 1, 27.1% (n = 13/48) of the patients in Group 2, 43.2% (n = 21/48) of the patients in Group 3 did not progress. Patients without progression were admitted on a median of 4 (2–19) days, while the other patients were admitted within 6 (1–17) days. We found that patients who applied early to the hospital had a significant "No Progress" status among all study groups (*p* = 0.040). When symptomatic improvement was evaluated, the mean was 4.16 (± 2.74) days in Group 1 and 5.59 (± 4.36) days in Group 2. A result close to statistical significance was obtained between these two groups (*p* = 0.059).

### Side effects and adverse reactions after treatment

In the examinations taken after drug use, the AST value changed less in Group 3 compared to Group 1 and 2; The *p* values for the comparison with Group 1 and Group 2 were < 0.001 and 0.022, respectively. Group 1 and 2 showed similar changes within themselves (*p* = 0.952). There was no significant increase in these laboratory values during follow-up. Serum creatinine and ALT changes were not statistically different between all groups. In the clinical presentation, two patients in Group 1 had diarrhea, and it resolved spontaneously within 48 h. Vomiting and nausea in 3 patients in Group 2. One patient in Group 3 had a feeling of chest palpitations, but ECG changes were not observed.

## Discussions

We conducted a retrospective study that FPV would be an effective drug against COVID-19 and would accelerate the clinical recovery of fever, cough, and breathing difficulties compared with patients who didn't take any antiviral treatment. In our study timeline, firstly, FPV was used in the treatment of patients whose clinics worsened while receiving HQ treatment or patients whose pneumonia findings progressed as guidelines suggested [8]. After updating the guidelines, it was recommended that patients start treatment early in combination with HQ as soon as they are diagnosed. Studies demonstrated that FPV is effective in slowing disease progression and viral clearance in COVID-19 as it is useful in Influenza and Ebola [11–13].

At the beginning of the pandemic, Lopinavir/Ritonavir, which was one of the drug combination considered as a promising antiviral drug for COVID-19, is not preferred today due to the intense drug interactions and lack of evidence that it reduces mortality [14]. In a study, FPV was found to have a greater impact on viral clearance than LPV/RTV [6]. In our study, it may seem that HQ alone provided better PCR negative results in Group 3 compared to Group 2. At the time, according to the Turkish Ministry of Health's Scientific Committees, FPV was considered as a second line treatment agent for patients who had the progressive disease under HQ treatment. Therefore, this discrepancy could be attributed to the fact that patients in Group 2 had more severe disease than Group 3. Another important finding was that patients in Group 1 had the best PCR negativity results. It may be that these participants benefitted from the early addition of FPV to HQ which was the suggested treatment at the time by the Turkish Ministry of Health's Scientific Committees’ guidelines. A randomized controlled study in China measuring the efficacy of FPV in COVID-19 compared to umifenovir, reported no significant difference between drugs [15]. However, the time to improvement in patients was shorter in the group treated with FPV [15]. Another effective therapy for considered for COVID-19 was Remdesivir [16]. On the contrary, a study reported Remdesivir treatment had no positive effect on mortality [17]. Due to their acceptable side effect profiles, both Favipiravir and Remdesivir continue to be used in many countries.

The most common symptoms at presentation in the study groups were cough (%61.8), myalgia (%56.9), fever (40.3%), and shortness of breath (40.3%), respectively. When Groups 1 and 2 were compared among themselves, it was found that early initiation of the drug led to early symptomatic improvement. Among all groups evaluated both radiologically and clinically, "No Progression" was more common in Group 1. These situations were interpreted as one of the beneficial results of early medication.

In a study, the median incubation time of COVID-19 was measured as 5.1 days [18]. However, our study reported the mean application time of the patients was 3.7 ± 2.5 days. The average time of admission to the hospital was shorter because some of the contact patients were referred to our hospital by filiation teams. All patients in Group 1 and 2 were symptomatic, but 13 (27.1%) patients in Group 3 were admitted to the hospital asymptomatically. It is known that the disease onset predicted in COVID-19 is within ten days in 95% of patients, and severe disease commonly present as ARDS typically begin 8–14 days after symptom onset [19]. Graselli et al., revealed that the rate of hospitalization in intensive care was 16% in patients who applied to the Lombardy region of Italy, which is one of the countries where the pandemic was felt most intensely, in the first two weeks [20]. Above this rate, it was observed that 27 (18.8%) patients were admitted to intensive care, and 6 (4.2%) patients were intubated. We have 6 (12.5%) patients in Group 1 and 10 (20.8%) patients in Group 2 who developed ARDS. No clinical progression was developed in the patients in Group 3.

In COVID-19 mortality rates may vary depending on the condition of the patients and the burden on the health system. In our study, the mortality rate among all patients was found lower than the rates in the literature [21]. This may be because our hospital did not encounter excessive occupancy in intensive care units during the process. However, our study reports a patient group with similar morbidity rates to the literature. It is known that comorbid diseases accompanying patients are the most important causes of death [22]. In our study, at least one comorbid disease was detected in 59 (40.9%) patients. The comorbid disease was present in 5 (83.3%) of the patients in our study who died. In the study, the incidence rates of the major diseases that make up this rate were reported similar to the literature for diabetes (17.4%), hypertension (31.3%), and cardiovascular (10.4%) disease [23]. The majority of patients (75%) in this group had moderate to severe clinical conditions.

High CRP and serum ferritin, known as poor prognostic factors shown in studies [24, 25], significantly decreased in patients that using FPV (Group 1 and 2). This indirect route may indicate that the use of FPV may have a good prognosis in the clinical course of patients. However, in the Kaplan–Meier analysis comparing the onset times of FPV treatment with survival, early treatment with FPV didn't provide any survival improvements. This situation may be related to the low number of our patients and the presence of many factors affecting mortality.

In a study comparing FPV with lopinavir (LPV)/ritonavir (RTV), significantly fewer side effects were observed in patients in the FPV arm [6]. In our study, ECG changes shown in previous studies [26, 27] such as the increased risk of prolongation of QT interval, drug-related "torsades de pointes" and sudden cardiac death were not observed in all groups. Although there was no significant change in ALT values in Group 1 and 2 compared to Group 3, the AST value in the patients using FPV (Group 1 and 2) was increased compared to Group 3. It is not certain whether it is secondary to COVID-19 disease or to the underlying disease. In our study, none of the patients had progressive deterioration in liver functions. Besides, improvement in the liver function tests without dose adjustment made it difficult for us to associate these disorders with FPV. There were no systemic or cutaneous adverse reactions observed in study groups. Consequently, controlled studies are needed to clarify the safety and side effect profile of FPV as a drug.

## Conclusion

The SARS-CoV-2 infection is spreading rapidly everywhere, and the lack of a cure for treatment in the world is seen as the biggest concern. According to the results of our study, we investigate clinical outcomes that will occur when FPV treatment is initiated early might enhance antiviral effects and improve clinical outcomes. We found data shows that early initiation of FPV as recommended will have positive consequences, but the results are needed to be broadly randomized and it should be verified by controlled studies. It will be the subject of further studies that FPV may have positive contributions to the treatment of COVID-19 in terms of viral clearance and mortality.

### Limitations

There were some limitations in this study. Firstly because it was conducted as a retrospective single center study our sample size was small. Secondly, as our knowledge and understanding of COVID-19 progressed over time our treatment options changed as well, resulting in different management preferences. Despite these, we believe this study is important as it is the largest nationwide real-life study to our knowledge that examines the clinical effectiveness of HQ and FPV drugs.

## Data Availability

Available upon reasonable request from the corresponding author.

## Abbreviations

COVID-19: Coronavirus disease 2019
FPV: Favipiravir
HQ: Hydroxychloroquine
WHO: World Health Organization
CRP: C-reactive protein
ARDS: Acute respiratory distress syndrome
CBC: Complete blood count;
CO-RADS: COVID-19 reporting and data system
RT-PCR: Reverse transcription PCR
CT: Computed tomography
LPV/RTV: Lopinavir/ritonavir
